# Using O*NET Linkages to Advance Research: An Example Evaluating Cognitive Function and Work Transitions

**DOI:** 10.1093/geroni/igab046.887

**Published:** 2021-12-17

**Authors:** Dawn Carr

**Affiliations:** Florida State University, Tallahassee, Florida, United States

## Abstract

The type of work older adults engage in has potential to play a key role in shaping health and wellbeing. In this presentation, using data drawn from an O*NET crosswalk linked with the Health and Retirement Study, I show how different types of transitions out of the workforce shapes cognitive function differently for individuals retiring from different types of occupations. Based on a factor analysis of 36 job-related abilities, activities, and contexts, this paper shows that retirement has a more significant consequence for cognitive function for those who retire from jobs with low levels of cognitive complexity, but no significant consequences for those who retire from jobs with high levels of cognitive complexity. I discuss these results in the context of the ways in which O*NET classifications of jobs can provide critical insights into the potential influence of changing retirement trajectories on wellbeing in later life.

